# Caspase-3 Is Involved in the Signalling in Erythroid Differentiation by Targeting Late Progenitors

**DOI:** 10.1371/journal.pone.0062303

**Published:** 2013-05-02

**Authors:** Daniela Boehm, Christelle Mazurier, Marie-Catherine Giarratana, Dhouha Darghouth, Anne-Marie Faussat, Laurence Harmand, Luc Douay

**Affiliations:** 1 Université Pierre et Marie Curie - Paris 6, UMR_S938 CDR Saint-Antoine, Prolifération et Différentiation des Cellules Souches, Paris, France; 2 INSERM, UMR_S938, Prolifération et Différentiation des Cellules Souches, Paris, France; 3 Etablissement Français du Sang Ile de France, Unité d'Ingénierie et de Thérapie Cellulaire, Créteil, France; 4 IFR 65-St Antoine, Université Pierre et Marie Curie - Paris 6, Plateforme de Cytométrie, Paris, France; 5 Assistance Publique - Hôpitaux de Paris, Hôpital St Antoine et Hôpital Trousseau, Service d'Hématologie Biologique, Paris, France; French Blood Institute, France

## Abstract

A role for caspase activation in erythroid differentiation has been established, yet its precise mode of action remains elusive. A drawback of all previous investigations on caspase activation in *ex vivo* erythroid differentiation is the lack of an *in vitro* model producing full enucleation of erythroid cells. Using a culture system which renders nearly 100% enucleated red cells from human CD34^+^ cells, we investigated the role of active caspase-3 in erythropoiesis. Profound effects of caspase-3 inhibition were found on erythroid cell growth and differentiation when inhibitors were added to CD34^+^ cells at the start of the culture and showed dose-response to the concentration of inhibitor employed. Enucleation was only reduced as a function of the reduced maturity of the culture and the increased cell death of mature cells while the majority of cells retained their ability to extrude their nuclei. Cell cycle analysis after caspase-3 inhibition showed caspase-3 to play a critical role in cell proliferation and highlighted a novel function of this protease in erythroid differentiation, i.e. its contribution to cell cycle regulation at the mitotic phase. While the effect of caspase-3 inhibitor treatment on CD34^+^ derived cells was not specific to the erythroid lineage, showing a similar reduction of cell expansion in myeloid cultures, the mechanism of action in both lineages appeared to be distinct with a strong induction of apoptosis causing the decreased yield of myeloid cells. Using a series of colony-forming assays we were able to pinpoint the stage at which cells were most sensitive to caspase-3 inhibition and found activated caspase-3 to play a signalling role in erythroid differentiation by targeting mature BFU-E and CFU-E but not early BFU-E.

## Introduction

Caspases, a group of highly conserved cysteine proteases which cleave specifically after an aspartate residue, play decisive roles in inflammatory and apoptotic processes but have also been implicated in non-apoptotic vital processes including cell differentiation, cellular remodelling and cell signalling [1], [2], [3], [4]. A significant function of caspases in erythropoiesis has been suggested by a number of investigations [5], [6], [7], [8] and the final stages in erythropoiesis have even been termed ‘aborted apoptosis’ due to numerous similarities including chromatin condensation, organelle removal and cell shrinkage without resulting in cell death. The involvement of caspases in erythroid differentiation was first established by Zermati *et al* [8], who detected their activation in *in vitro* erythroid cultures and reported a block of differentiation at the basophilic erythroblast stage upon caspase inhibition. It has since been shown that caspase-3 is transiently activated in the first 8 days of CD34^+^ cell-derived erythroid culture and erythroid maturation is reduced by siRNA against caspase-3 [5]. Carlile *et al* linked the pro-differentiative effect of caspase activation in erythroid cells to the activation of the Fas receptor on CD34^+^ cells and found that silencing of FasR resulted in a similar block of differentiation as silencing of caspase-3 expression [9].

While a transient non-apoptotic activation of caspases seems established in *ex vivo* erythroid systems, questions remain regarding the cause of this activation, the cellular targets and whether this activation is essential for erythroid enucleation. Despite the majority of cellular changes (enucleation, loss of mitochondria and organelles, membrane restructuring) occurring in late stage erythropoiesis, no concurrent caspase activation has been found and the activation of caspase-3 appears to be limited to the early stages of culture [6], [8], [9]. Studies of knock-out mice lacking caspases−1,−2,−3 and−9 also showed no evident abnormalities in the generation of red blood cells [10]. The precise role of caspases in normal erythroid development thus remains elusive. In an attempt to shed light on these controversies we used a highly proliferative *in vitro* erythropoiesis model that renders nearly 100% enucleated cells which have been shown to be functional *in vitro* and *in vivo* both in animal models and in human [11], [12]. This *ex vivo* system has been shown to be a powerful tool for the fundamental study of erythropoiesis in a physiological and pathological context [13].

Using this model, we characterized the effect of caspase-3 inhibition on erythroid cell expansion, viability and differentiation, investigated the stage at which erythroid cells show highest susceptibility to caspase-3 inhibition and assessed for erythroid-specificity by comparing it to the myeloid differentiation system. We show here that caspase-3 inhibition does not specifically prevent terminal maturation, i.e. erythroid enucleation, but plays an important signalling role in early erythroid differentiation. Through a series of clonogenic assays, we were able to specify the stage in erythroid development at which cells are most susceptible to the inhibition of caspase-3, showing that the later type progenitors BFU-E and CFU-E are sensitive to this inhibition, while the earliest progenitors remain unaffected.

## Materials and Methods

### Cell cultures

CD34^+^ cells were isolated from cord blood (CB) samples by immunomagnetic separation using anti-CD34 beads and MACS columns (Miltenyi, Bergisch Gladbach, Germany). Cord blood was collected by the public cord blood bank of EFS Ile de France in Creteil which is authorized by the French regulatory agency (ANSM) with the n° TCG/10/R/003. Informed consent was signed by all patients before the CB collection according to the French cord blood registry (accredited WMDA).

#### Erythroid cultures

Erythroid cultures were expanded in erythroid differentiation (EDM) medium as previously published [12]. Briefly EDM was composed of IMDM (Iscove's modified Dulbecco's medium, Biochrom, Berlin, Germany) containing 1% of stabilized glutamine, and was supplemented with 330 µg/ml iron-saturated human transferrin (Scipac, Sittingbourne, UK), 107 g/ml recombinant human insulin (Sigma, Saint-Quentin Fallavier, France), 2 IU/ml heparin (Sanofi, France) and 5% of human plasma (Etablissement Français du Sang, France).

EDM was supplemented with 100 ng/ml Stem Cell Factor (SCF), 5 ng/ml Interleukin-3 (IL-3) (PeproTech, Neuilly-sur-Seine, France) and 3 IU/ml erythropoietin (EPO) (Eprex, kindly provided by Janssen-Cilag, Issy-les-Moulineaux, France) in the first 11 days of culture and solely with EPO thereafter. Cells were seeded at 1×10^4^ cells/ml on day 0, diluted 1 in 5 in fresh medium on day 4 and were reseeded in fresh medium at 5×10^4^ cells/ml on day 7 or 8, at 7×10^5^ cells/ml on day 11, at 4×10^6^ cells/ml on day 14 and 10×10^6^ cells/ml on day 18.

Cultures were supplemented with the caspase-3/7 inhibitor 5-[(S)-(+)-2-(Methoxymethyl)-pyrrolidino]-sulfonylisatin (SIT) (Santa Cruz Biotechnology, Santa Cruz, CA, USA). SIT is a synthetic, non-peptidic inhibitor that binds to a site adjacent to the enzyme's active centre in a reversible manner [14], [15]. SIT was reconstituted at 100 mM in DMSO (Sigma) and used at a final concentration of 50 µM (except for dose response determination where concentrations varied from 25 µM to 200 µM). Controls consisted of cultures supplemented with corresponding concentrations of DMSO, i.e. 0.05% for a corresponding dose of 50 µM of SIT.

Cultures were supplemented with fresh inhibitor or DMSO at day 0 and at each time point of culture dilution/reseeding and shielded from light when supplemented with caspase inhibitor. Cells were cultured in T25 flasks or multi-well plates (Becton Dickinson, Le Pont de Claix-Cedex, France) at 37°C, 5% CO_2_ in a humidified incubator.

Cell enumeration and viability assessments were performed using trypan blue exclusion staining and May-Grünwald Giemsa (RAL diagnostics, Martillac, France) staining was carried out for morphological analyses.

#### Myeloid cultures

Myeloid cultures cells were cultured in EDM medium supplemented with 100 ng/ml SCF, 5 ng/ml IL-3, and 20 ng/ml Granulocyte Colony Stimulating Factor (G-CSF) (PeproTech).

Cells were seeded at 5×10^3^ cells/ml on day 0 and expanded cells were diluted 1 in 4 in fresh cytokine-supplemented medium at day 6. Cultures were supplemented with 50 µM of SIT (except for dose response determination where concentrations varied from 25 µM to 200 µM) or 0.05% DMSO on day 0 and day 6.

Cells were cultured in T25 flasks or multi-well plates (Becton Dickinson) at 37°C, 5% CO_2_ in a humidified incubator and shielded from light when supplemented with caspase inhibitor.

Cell enumeration and viability assessments were performed using trypan blue exclusion staining and May-Grünwald Giemsa (RAL diagnostics) staining was carried out for morphological analyses.

### Methylcellulose-based colony forming assays

Growth factor supplemented methylcellulose (Stem Cell Technologies, Grenoble, France) containing SCF (50 ng/ml), G-CSF (20 ng/ml), Granulocyte Macrophage Colony Stimulating Factor (GM-CSF) (20 ng/ml), IL-3 (10 ng/ml) (Peprotech) and EPO (3 IU/ml) (Janssen Cilag) was used for haematopoietic colony-forming assays. CD34^+^ cells were seeded at 500 cells per plate on day 0.

Alternatively, CD34^+^ cells were first grown in suspension culture supplemented either with SCF, IL-3 and EPO or with SCF, IL-3 and G-CSF. Cells from these cultures were then seeded in methyl-cellulose containing either 0.05% DMSO or 50 µM SIT every day for the first 7 to 10 days of cultures. 500 to 2500 cells were seeded per plate.

CD34^low^ and CD34^neg^ fractions were seeded at 500 to 1000 cells per plate in methyl-cellulose containing either 0.05% DMSO or 50 µM SIT.

After 7 and 14/18 days at 37°C and 5% CO_2_, colonies were scored as follows:

At day 7, CFU-E were identified as very small single units (less than 100 cells per clone) which were haemoglobinized. At day 14/16 early BFU-E consisted of very large colonies composed of haemoglobinized sub-units or one very large cluster, while mature BFU-E were colonies with a more restrictive size. Myeloid progenitors (CFU-G, CFU-GM or CFU-M) were scored at day 14 and were identified as non-haemoglobinized colonies.

### Immunoprecipitation and blot analysis

For immunoprecipitation of caspase-3, 2×10^7^ Jurkat cells or erythroid cells from day 7 of culture were washed with ice-cold PBS and lysed in 400 µl of Lysis buffer A (10 mM HEPES [pH 7.6], 3 mM MgCl_2_, 10 mM KCl, 5% glycerol, 0.5% NP-40) containing phosphoserine/threonine phosphatase inhibitors (20 mM NaF, 1 mM sodium pyrophosphate, 25 mM β-glycerophosphate), and protease inhibitor cocktail (Roche) for 30 min at 4°C. Lysates were cleared by centrifugation at 14,000×*g* for 10 min at 4°C. After centrifugation 200 µl of lysate were incubated with 1 µg of anti-active caspase-3 antibody (n° 559565, BD Pharmingen) at 4°C on a rotating wheel over night prior to addition of 25 µl of protein A/G Plus-agarose coupled microbeads (SC-2003, Santa Cruz Biotechnology) and an additional incubation for 1 hour on rotation at 4°C. A control immunoprecipitation was performed in parallel, using 4 µg of non-immune normal rabbit IgG (Jackson Immunoresearch) and 200 µl of lysate.

The protein A/G Plus-agarose beads with bound immune complexes were washed four times with buffer A and resuspended in Laemmli buffer. Proteins were separated by NuPAGE® Novex® 10% Bis-Tris Gel (Invitrogen) and transferred to a nitrocellulose membrane (Invitrogen). The blot was blocked with 5% nonfat milk in Tris-buffered saline (TBS)−0.1% Tween-20 and incubated with anti-total caspase-3 (n° 9662, Cell Signaling) (1∶500 dilution). The membrane was washed four times in TBS−0.1% Tween 20 and incubated for 1 hour with the anti-rabbit horseradish peroxidase-conjugated secondary antibody (n°7074, Cell Signaling) (1∶10000 dilution). Antigens were visualized by chemiluminescence using SuperSignal West Pico (Pierce).

### Cell cycle analysis

Cells from day 7 of the erythroid or myeloid culture were adjusted at 10^6^ cells/ml and treated with 50 µM SIT (Santa Cruz). Cells were harvested at 2 h, 4 h, 6 h, 8 h and 24 h after the beginning of the treatment. Controls consisted of cultured cells supplemented with 0.05% DMSO (Sigma). Treated and untreated cells were subjected to DNA content analysis. Briefly, 10^6^ cells were washed twice with PBS and fixed with 1 ml of cold 70% ethanol added dropwise, vigorously vortexed and then kept at−20°C for at least 24 h. Cells were pelleted and ethanol was removed before 2 abundant washes in PBS. DNA was stained by incubating the cells in 600 µL of PBS containing 5 µg propidium iodide (PI) (Becton Dickinson), 50 µg RNase A (Fermentas Thermo Fisher Scientific, Villebon sur Yvette, France) and 0.15% Igepal CA-630 (Sigma). The cells were incubated in the dark for 10 min at 37°C and cell cycle analysis was performed within 1 hour using a BD LSR II flow cytometer (Becton Dickinson). 10^4^ events were analysed for each sample and doublets were excluded from the analysis. Data were analysed using Modfit software (Becton Dickinson).

### Flow cytometric analysis

Fluorescein isothiocyanate (FITC) or phycoerythrin (PE) conjugated antibodies against CD34, CD36, CD13, CD45, CD71 and Glycophorin A (GlyA) (all from Beckman Coulter, Marseille, France) and corresponding isotype controls were used for surface antigen labelling and cells were analysed on a FACSCalibur (Becton Dickinson) or Cyan ADP Analyzer (Beckman Coulter) flow cytometer. Apoptotic/necrotic cells were determined using staining with AnnexinV-FITC and Propidium Iodide (PI) with the Annexin V staining kit (Beckman Coulter) according to the manufacturers' instructions.

### Calcein AM viability test

The calcein test was performed according to the procedure of Bratosin *et al* [16]. Briefly, 2×10^5^ RBC were incubated in the presence of 5 µM calcein AM (Sigma) in a final volume of 200 µL for 45 min at 37°C under aerobiosis. The cells were then resuspended in 500 µL of PBS (pH 7.4) and analysed immediately by flow cytometry. A control without calcein AM was performed under the same conditions. A cell was considered to be viable if the intensity of the fluorescence was superior to 10^2^ on an arbitrary scale ranging from 1 to 10^4^.

### Fluorescence-activated cell sorting

The CD34^low^ and CD34^neg^ fractions were sorted using a FACS MofloAstrios (Beckman Coulter) equipped with Summit software (Beckman Coulter). Cells were labelled with 20 µL APC-CD34 antibody (Beckman Coulter) per 1×10^6^ cells. Cells were collected in IMDM medium supplemented with 20% of FCS (Dominique Dutscher, Illkirch, France), then washed in PBS before cultivation in semi-solid methylcellulose medium as described above.

### Statistical analysis

All data are expressed as mean ± standard error of the mean (SEM). Statistical comparisons were made using paired Student's t-tests. A p-value of<0.05 was considered significant.

## Results

Using different cytokine combinations, we investigated the effect of caspase-3 inhibition at different stages of erythroid culture in order to clarify how caspase-3 activation modulates erythroid differentiation, a role that is so far not fully understood. We analysed the effects on production, expansion and differentiation of erythroid progenitors and precursors and compared these results to a system promoting myeloid differentiation.

### Caspase-3 inhibition reduces *in vitro* erythroid proliferation and delays erythroid differentiation

**Figure 1:**
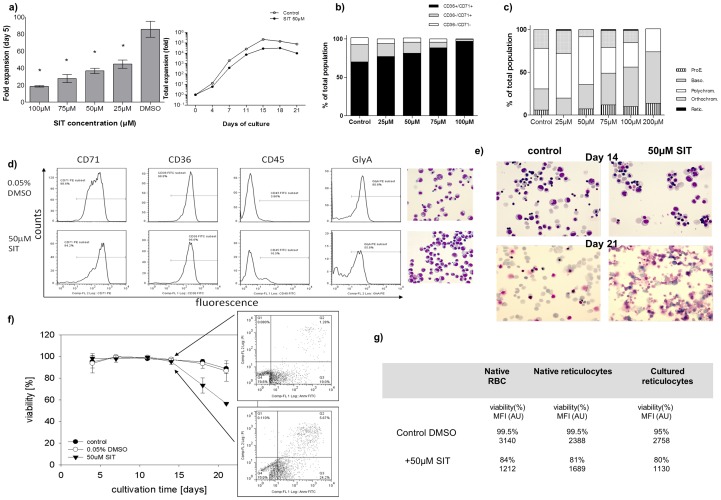
Caspase-3 inhibition affects erythroid proliferation, differentiation and viability. **a) Left: Dose-response of SIT treatment on erythroid cell expansion (day 5).** Reduction of cell expansion as function of the inhibitor concentration (results shown as mean ± SEM, n = 3 experiments). All inhibitor concentrations showed significantly reduced expansion compared to control (* p<0.05) and also differed significantly (p<0.05) from each other (exception 50 µM to 25 µM). **Right: Effect of caspase-3 inhibitor treatment on expansion of CD34^+^-derived erythroid cultures.** Expansion curves of erythroid cultures under control (0.05% DMSO, open circles) or SIT-treated (filled circles) conditions showed growth reduction when caspase-3 is inhibited. **b and c) Dose-response of erythroid differentiation to caspase-3 inhibitor treatment.** Expression of surface antigens CD36 and CD71 in erythroid culture (day 13, b) and distribution between stages of erythroid differentiation (day 11, c) according to SIT treatment (one representative experiment shown). Proerythroblast (ProE), basophilic erythroblast (Baso), polychromatic erythroblast (Polychrom), orthochromatic erythroblast (Orthochrom), reticulocyte (Retic). **d) Erythroid phenotype after caspase-3 inhibitor treatment.** Almost all cells (day 11) were erythroid (CD36^+^/CD71^+^) but delayed differentiation was apparent under SIT treated conditions (bottom) compared to control (top) in respect to expression of surface markers CD71, CD45 and GlyA as assessed by flow cytometry (one representative experiment shown). **e) Erythroid enucleation after caspase-3 inhibitor treatment.** Enucleation progressed comparably at day 14 (upper panel) in cultures treated with 50 µM SIT (right) or DMSO (left) but a high degree of cell lysis was observed after day 21 (lower panel) in SIT treated cultures (right) compared to DMSO treated control (left) (one representative experiment shown). **f) Reduction of viability in late stages of culture.** Viabilities up to day 14 did not differ significantly between 50 µM SIT and controls (0.05% DMSO or untreated) but differed significantly (p<0.05) from control on day 18 and from control and DMSO on day 21 as determined by trypan blue exclusion. Flow cytometry analysis using AnnexinV-FITC and PI on day 14 showed higher percentages of early apoptotic (AnnexinV^+^) and late apoptotic (AnnexinV^+^/PI^+^) cells in SIT treated cultures (inset, bottom) vs. control (inset, top) (one representative experiment shown). **g) Effect of caspase-3 inhibitor on mature cells.** The effect of SIT was tested on native red blood cells (RBC), native reticulocytes and cultured reticulocytes using the calcein AM viability assay. Results are expressed as mean fluorescence intensity (MFI) or percent of cells (%) (one representative experiment shown).

The supplementation of erythroid cultures with the caspase-3 inhibitor SIT resulted in a significant reduction of cell expansion, showing dose-response between 25 and 100 µM (**Figure 1a** **, left**), with 200 µM basically inhibiting any determinably proliferation while corresponding concentrations of DMSO solvent control did not evoke any significant growth reduction. The inhibitor was subsequently used primarily at a concentration of 50 µM as this resulted in an overall growth reduction of 50–80% (**Figure 1a** **, right**) without showing significantly increased cell death until day 15 of culture.

Caspase-3 inhibition also reduced erythroid differentiation in a dose dependent manner with cells being less differentiated with increasing concentrations of SIT. Inhibitor treated cultures showed a slower progression from immature (CD36^−^) cells to committed erythroid CD36^+^ cells (results not shown). Delayed differentiation was visible in cell size, morphology and erythroid marker expression and maturation was equally delayed in a dose-dependent manner as shown by the slower disappearance of CD71 and CD36 at day 13 (**Figure 1b**). At day 11 inhibitor-treated cultures still contained higher numbers of proerythroblasts and basophilic erythroblast (**Figure 1c**) compared to controls and the use of 200 µM SIT, while resulting in minimal cell expansion, also inhibited any differentiation past the polychromatophilic stage. Phenotypically, we furthermore observed the persistence of the haematopoietic marker CD45 and a reduced mean fluorescence intensity (MFI) of glycophorin A for inhibitor-treated cells compared to controls (**Figure 1d**). The treatment with a caspase-3 inhibitor did not appear to inhibit enucleation *per se* but higher numbers of nucleated cells could be observed in treated cultures as a result of generally reduced differentiation and increasing cell death of more mature cells. At high concentrations of inhibitor the cells became arrested at an early erythroblast stage whereas lower concentrations allowed progression to the orthochromatic erythroblast or reticulocyte stage. While enucleation was comparable between cultures treated with 50 µM SIT and control at day 14, high cell death/lysis (day 21) was found to prevent treated cultures from yielding higher numbers of enucleated cells thereafter (**Figure 1e**).

### Caspase-3 inhibition increases cell death in late stages of culture

Caspase-3 inhibition reduced expansion of erythroid cells derived from CD34^+^ without showing increased cell death either during the amplification or differentiation of erythroid progenitors/precursors (between day 0 and day 15).

However, while early stages of culture showed no significant differences in cell death between treated and non-treated cells, higher numbers of AnnexinV^+^ cells were detected from the orthochromatic erythroblast stage onwards along with a sharp decrease in overall culture viability and rupture of almost all cells after day 21 (**Figure 1f**).

Directly testing SIT on more mature cultured reticulocytes in late stages of the culture (day 18) revealed that the inhibitor weakly diminishes cell viability as demonstrated by the calcein viability test. We compared the mean fluorescence intensity (MFI) of the calcein dye for native RBC, native reticulocytes sorted by CD71 immunoselection and cultured reticulocytes, and observed, that after 3 days of incubation with SIT, calcein MFI and positivity were reduced to the same level for all types of cells (**Figure 1g**). This suggests that enucleated cells might be more sensitive to a potential toxic effect of the inhibitor.

### Caspase-3 inhibition in the erythroid lineage does not lead to apoptosis but delays cell cycle progression through the G_2_/M phase

**Figure 2:**
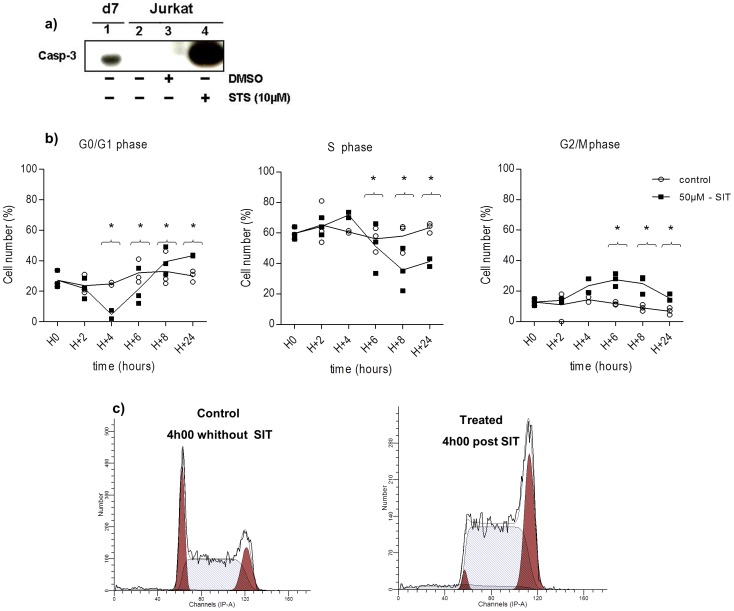
Caspase-3 inhibition modulates cell cycle progression in the erythroid lineage. **a) Immunoprecipitation of activated caspase-3 from cell lysates of cultured erythroid cells at day 7.** Whole cell lysate from 10×10^6^ cells was immunoprecipitated with 1 µg of the rabbit anti-active caspase-3 antibody. Lane 1: erythroid cells at day 7 of culture (d7), lane 2: untreated Jurkat cells, lane 3: Jurkat cells incubated with DMSO for 18 h and lane 4: Jurkat cells treated with 10 µM staurosporin (STS) for 18 h. The activated 23 kDa caspase-3 band seen in control Jurkat cells induced into apoptosis by STS is clearly visible in erythroid cells (lane 1). **b) Cell cycle analysis on day 7 of cultured erythroid cells.** Cells were treated with SIT 50 µM or not and harvested after 2, 6, 8 and 24 hours. Controls consisted of cultured cells supplemented with 0.05% of DMSO. Controls are plotted as empty bullets, treated cells are plotted as black squares. Connecting curves represent mean of results. * indicates a statistically significant result with p<0.05 according to a paired t-test. 3 independent experiments were performed and are plotted on one graph. **c) Caspase-3 inhibitor delays progression through G_2_/M phase in erythroid cultures.** Representative histograms of cell cycle profiles of cells treated with 50 µM SIT or controls (0.05% DMSO), 4 h after SIT or DMSO treatment.

The activation of caspase-3 has been reported as transient and effective after 5 to 7 days of a culture stimulated by EPO, SCF and IL-3. We subsequently focused on day 7 of erythroid culture and confirmed the activation of caspase-3 by immunoprecipitation (**Figure 2a**). In order to explain the observed reduction in cell expansion after caspase-3 inhibition, we investigated the cell cycle progression between 2 hours and 24 hours after SIT treatment by flow cytometry. The kinetic of the cell cycle progression for treated and non-treated cells is shown in **Figure 2b**.

In steady state, cells were mainly in S phase (59.7%±0.04), while 27.2%±0.06 of cells were in G_1_ phase and 12.8%±0.02 were in G_2_/M phase. Controls and SIT treated cells showed the same cell cycle profile until 4 hours post treatment. At this time of the kinetic, we observed a significant decrease of cells with G_1_ DNA content (mean of 80%, p = 0.05) and to a lesser extent an accumulation in G_2_/M phase (23.7%±0.06 in SIT-treated cells vs. 14.5%±0.02 in controls) in SIT treated cultures. This breaking down in G_1_ phase subsequently faded but an increase (mean of 2.5 fold) in the percentage of SIT treated cells with G_2_/M DNA content became apparent from 6 hours post-treatment onwards (at 6 h post treatment, 27.5%±0.04 vs. 11.8%±0.01, p = 0.01, at 8 h post treatment, 24.9%±0.06 vs. 9%±0.02, p = 0.03, at 24 h post treatment, 15.4%±0.02 vs. 6.8%±0.02, p = 0.04). A representative cell cycle histogram plot 4 hours after treatment is given in **Figure 2c**.

We also observed a significant decrease (mean of 0.73 fold) in the percentage of SIT treated cells containing S-phase (>2N) DNA content (at 6 h post treatment, 51.2%±0.2 vs. 56.2%±0.08, p = NS, at 8 h post treatment, 35.7%±0.14 vs. 58%±0.1, p = 0.01, at 24 h post treatment, 41.3%±0.03 vs. 63.5%±0.03, p = 0.08).

Interestingly, we did not observe a sub-G_1_ DNA accumulation in either SIT-treated or non-treated cells, confirming that inhibition of caspase-3 did not induce apoptosis in the erythroid lineage at this time of differentiation.

We conclude that caspase-3 inhibition in erythroid differentiation impairs normal cell cycle progression by delaying the progression through the G_2_/M phase. Stalling in G_2_ and/or a failure to correctly perform mitosis cause the drop in G_1_ phase and the subsequent decrease in S phase, as reduced numbers of cells progress to these stages, and explain at least in part the reduction of cellular expansion. This effect is rapidly observable (4 h after treatment) and observable at a lesser extend for 24 h.

**Figure 3:**
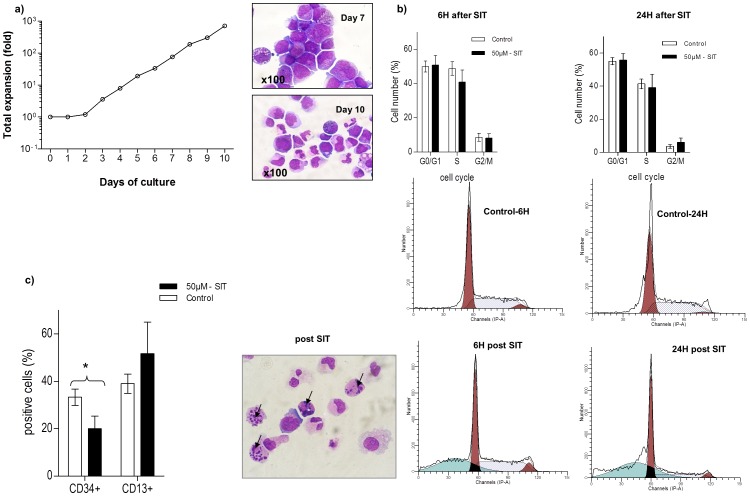
The inhibition of caspase-3 induces apoptosis in the myeloid lineage. **a) Myeloid expansion and differentiation.** Expansion curve of a myeloid culture (one representative experiment and photographs of the cells on days 7 and 10 of myeloid culture after May-Grünwald-Giemsa staining (X100)). **b) Cell cycle analysis on day 7 of cultured myeloid cells.** Cells were treated with SIT 50 µM or not and harvested after 6 and 24 hours. Controls consisted of cultured cells supplemented with 0.05% of DMSO. 3 independent experiments were performed. Controls are plotted as empty bars, treated cells are plotted as black bars. Results are expressed as mean ± SEM (3b, top). Representative histograms of cell cycle profiles for control (0.05% DMSO) and treated cells (50 µM SIT) 6 h and 24 h after treatment (3b, bottom). Results indicate that caspase-3 inhibition induced apoptosis in myeloid cultures. Photograph of treated cells (day 10) 24 h post treatment after May-Grünwald-Giemsa staining (X100) (3b, bottom left). **c) FACS analyses of CD13 and CD34 marker expression.** Expression of surface antigens CD13 and CD34 in myeloid culture (day 10) for both 50 µM SIT and control (0.05% DMSO). Results in percent (%) are expressed as mean ± SEM, n = 3; * indicates a statistically significant result with p<0.05 according to a paired t-test.

To test for a possible specificity of caspase-3 activation to the erythroid system, we translated experiments into a culture system which favours myeloid differentiation. Using a cocktail of SCF, IL-3 and G-CSF, we investigated the effect of caspase-3 inhibition at day 7 of myeloid differentiation. The obtained cells had mainly differentiated into myeloid cells, i.e. 76% at day 7 and 90% at day 10, the remaining cells were blast cells (24% at day 7, 10% at day 10) (**Figure 3a**). An obvious maturation was observed within the myeloid cells between day 7 and day 10 of the culture as stated in **Table 1**.

**Table 1:** Cytology of myeloid cells at day 7 and day 10.

Cytology (%)	Day 7	Day 10
Blast cells	24	10
Basophilic cells	6	5
Myeloblasts	16	4
Promyelocytes	38	18
Myelocytes	11	34
Metamyelocytes	4	13
Polymorphonuclear cells	1	16

A supplementation of the myeloid cultures with the caspase-3 inhibitor SIT resulted in a reduction of cell expansion by 35% at day 7/10 of the culture (data not shown).

When we studied the effect of the inhibitor on the cell cycle, we observed that blocking caspase-3 in myeloid cells induced apoptosis (6 hours post treatment a mean of 29.4% of cells were apoptotic vs. 4.8% in controls, 24 hours post treatment a mean of 25.9% of cells were apoptotic vs. 4.5% in controls), as shown by the accumulation in sub-G_1_ DNA content (**Figure 3b**). Only a minor effect was observed on the G_2_/M phase which was slightly increased in SIT treated cells (**Figure 3b**).

A block in differentiation of treated myeloid cells was not apparent but differentiation appeared accelerated instead. Indeed, at day 7 of the culture, the percentage of CD34^+^ cells in cultures supplemented with 50 µM SIT was lower than in control cultures (20%±5% vs. 33%±5%, p = 0.05), while one of the myeloid markers, CD13, was increased in SIT treated cultures (52%±13% vs. 39%±7%). We conclude that caspase-3 inhibition in myeloid differentiation does not impair cell cycle progression but rapidly leads to apoptosis, resulting in an overall reduction of cellular expansion (**Figure 3c**).

### Caspase-3 inhibition does not modulate the production of primitive erythroid progenitors but affects late progenitors

**Figure 4:**
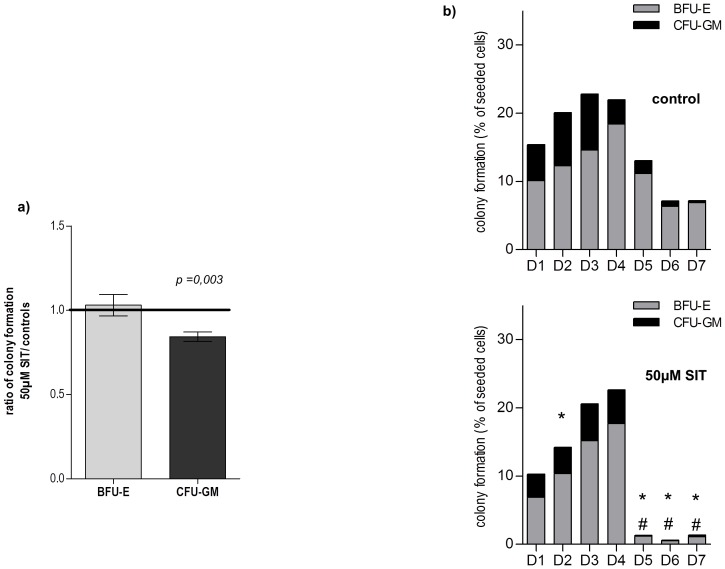
Caspase-3 inhibition affects the production of later erythroid progenitors but not primitive progenitors. **a) Effect of caspase-3 inhibition on CD34^high^ cells.** CD34^high^ were plated in methylcellulose colony-forming assays supplemented with DMSO or 50 µM SIT. Colony formation was assessed after 14/18 days. Results shown are the mean of 6 independent experiments and are expressed as mean ratio of colony formation in 50 µM SIT conditions to controls. * indicates a significant difference (p<0.05). **b) Caspase-3 inhibition in the erythroid lineage did not modulate the production of primitive progenitors but decreased the production of later progenitors.** Cells from each of the first 7 days of erythroid culture were plated in methylcellulose colony-forming assays supplemented with DMSO (top) or SIT (bottom). Colony formation was assessed after 14/18 days in respective conditions (results shown are the mean of duplicate plates from a pool of 3 cord blood units). Significant differences (p<0.05) in colony formation compared to control cultures are marked by # for BFU-E, * for CFU-GM.

The reduction of proliferative potential observed in both erythroid and myeloid liquid cultures led us to assess the effect of caspase-3 on cloning efficiency using methyl cellulose assays. For that purpose CD34^+^ cells selected by immunoselection (which correspond to day 0 of liquid culture) were treated with 50 µM SIT and seeded in methylcellulose. The cloning efficiency for BFU-E was found to be unaffected by inhibitor treatment (ratio of colony formation of SIT to control = 1.03±0.15; n = 6). Interestingly only the cloning efficiency of CFU-GM was diminished by 16% in inhibitor treated cultures (ratio of colony formation of SIT to control = 0.84±0.07, p = 0.003, n = 6) (**Figure 4a**). The inhibition of caspase-3 thus appears to diminish the number of myeloid progenitors CFU-GM, but not erythroid progenitors BFU-E.

As the immunomagnetic selection for the CD34 marker favours isolation of CD34^high^ haematopoietic stem/progenitor cells this type of experiment only allows to assess CD34^high^ (mean of CD34^+^ MFI = 921, n = 7) haematopoietic stem cells and consequently fails to correctly evaluate a potential effect of caspase-3 inhibition on more mature progenitors. In methylcellulose assays, this population of CD34^high^ is enriched in BFU-E but contains only few late erythroid progenitors such as CFU-E (less than 2% of the total CFC). In an attempt to determine at which point cells became sensitive to caspase-3 inhibition, colony-forming assays supplemented with 50 µM SIT (or 0.05% DMSO as controls) were subsequently launched on a daily basis from the first 7 days of an untreated erythroid liquid culture (as described above). The supplementation of the medium with EPO and the duration of the culture permitted us to analyse the effect of caspase-3 on primitive and more mature erythroid progenitors in this series of CFC assays.

Clonogenic cells contained in D0–D7 expanded control cells belonged mainly to the erythroid lineage, yet a considerable myeloid colony forming potential remained from D0 to D4 of expansion (43% at day 0 and 36% at day 4) and then dramatically diminished to represent 4% of the clonogenic cells at day 7.

For inhibitor treated cells derived from the first 4 days of culture, the cloning efficiency for BFU-E was unchanged when compared to control whereas the cloning efficiency of CFU-GM was diminished by a mean of 35% in inhibitor treated cultures.

From day 5 onwards we observed a very strong effect (reduction of total colony yield by 90%) on both erythroid and myeloid lineages (**Figure 4b**). We hypothesize that the inhibition of caspase-3 in the erythroid lineage does not modulate the production of primitive progenitors but decreases the production of later progenitors, whereas its effect is immediate on the myeloid lineage.

**Figure 5:**
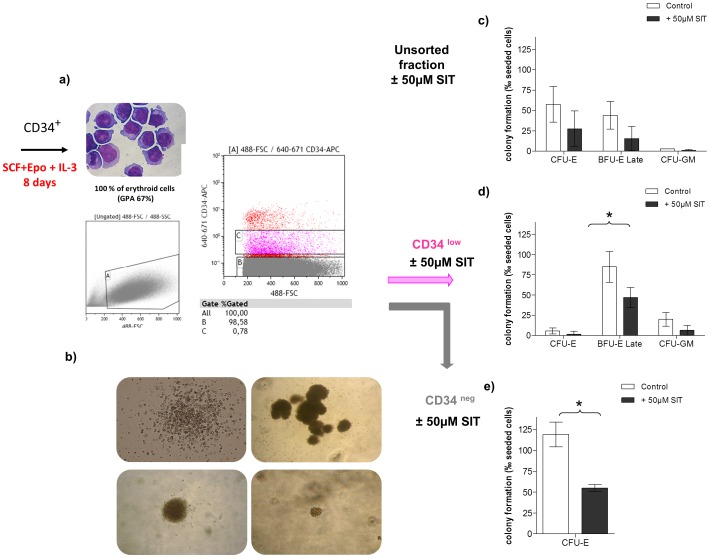
Caspase-3 inhibition decreases the production of late BFU-E and CFU-E. **a) CD36^+^/CD34^low^ and CD36^+^/CD34^neg^ sorted cells derived from 7 days of untreated erythroid liquid culture.** The CD34^low^ (pink) and CD34^neg^ (grey) fractions were sorted using a FACS MofloAstrios after labelling with APC-CD34 antibody. Cells were collected in IMDM medium+20% of FCS then washed in PBS before cultivation in semi-solid methylcellulose medium. **b) Different types of progenitors obtained in methyl cellulose assays. Top left**: a non-haemoglobinized colony scored at day 14 and identified as a myeloid progenitor. **Top right**: an early BFU-E consisting of a very large colony composed of haemoglobinized sub-units and scored at day 14/18. **Bottom left**: a mature BFU-E identified by its restricted size. **Bottom right**: a CFU-E identified as a very small single unit that is haemoglobinized at day 7 of the assay. **c, d, e) Caspase-3 inhibition in the erythroid lineage did not modulate the production of primitive progenitors but decreased the production of late BFU-E and CFU-E.** Cells from the first 7 days of erythroid culture (unsorted fraction (c), CD36^+^/CD34^low^ sorted cells (d) or CD36^+^/CD34^neg^ cells (e) were plated in methylcellulose colony-forming assays supplemented with DMSO (empty bars) or SIT (black bars). Colony formation was assessed after 14/18 days in respective conditions. * indicates significant differences (p<0.05) in colony formation compared to control cultures (paired t-test).

To test this hypothesis, we proceeded to analysing the effect of caspase-3 inhibition on mature erythroid progenitors (100% CD36^+^). To this end, cells from day 7 of an untreated erythroid liquid culture were sorted into CD34^low^ and CD34^neg^ populations (**Figure 5a**) (as described above), and seeded in methylcellulose in the presence or not of 50 µM SIT. **Figure 5b** depicts the different types of colonies that were obtained.

The CD34^low^ fraction contained mainly mature BFU-E, some myeloid progenitors CFU-GM and some CFU-E. A very strong effect of the caspase-3 inhibitor was observed on all types of progenitors, all of which were decreased. The action of the caspase-3 inhibitor diminished the formation of mature BFU-E by 45% (85%±11% in control vs. 47%±7.2% in SIT-treated culture, n = 3, p = 0.04); the CFU-GM formation by 68.5% (20%±4.9% in control vs. 6.3%±3.5% in SIT-treated culture, n = 3) and the CFU-E formation by 73% (5.5%±2.1% in control vs. 1.5%±2.1% in SIT-treated culture, n = 3) (**Figure 5d**).

The CD34^neg^ fraction contained only CFU-E. Caspase-3 inhibition showed a very strong effect on these very mature progenitors which were decreased by 54% (119%±8.5% in control vs. 55%±2.4% in SIT-treated culture, n = 3, p = 0.002).

These findings support our hypothesis that inhibition of caspase-3 modulates the production of more mature erythroid progenitors such as CFU-E and mature BFU- E (**Figure 5d**). The total fraction (unsorted) of cells, in which CD34^+^ expression was dramatically diminished (MFI = 53) compared to day 0 cells (MFI = 1470), showed similar results (**Figure 5a**).

We subsequently cultured CD34^+^ cells in the presence of a cocktail of SCF, IL-3 and G-CSF. This cytokine combination does not allow erythroid differentiation due to the lack of EPO but preserves immature erythroid progenitors (early BFU-E) while permitting myeloid differentiation in 7–10 days. Colony-forming assays launched on a daily basis from the first 10 days of liquid culture consequently did not contain very mature BFU-E or CFU-E.

**Figure 6:**
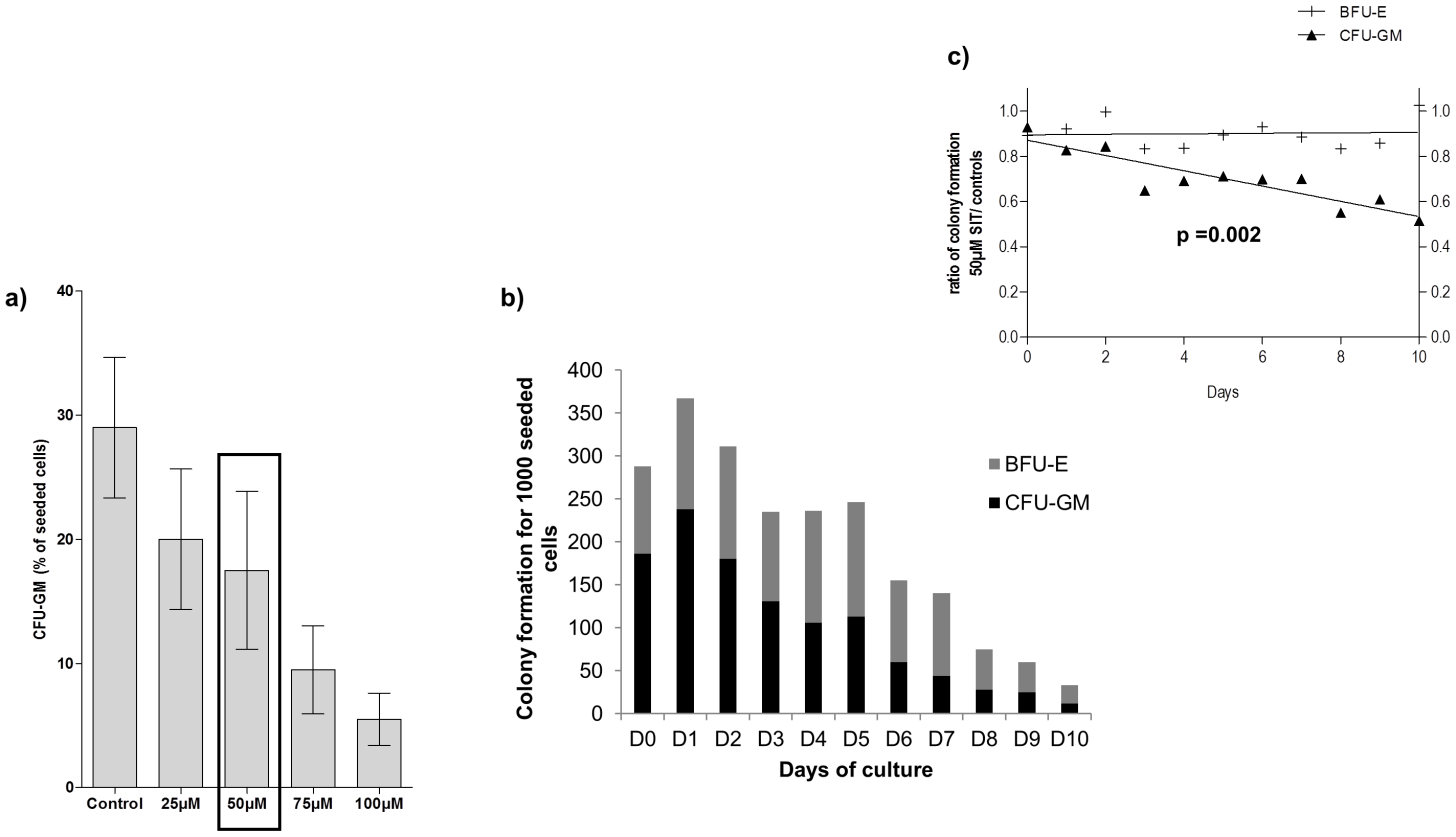
Caspase-3 inhibition does not affect the production of early BFU-E. **a) Dose-response of SIT treatment on CFU-GM formation.** Reduction of CFU-GM formation as function of the inhibitor concentration (results shown as mean ± SEM, n = 3 experiments). **b) The effect of caspase-3 inhibition on early BFU-E and CFU-GM.** Cloning efficiency of untreated cells for the first 10 days of a myeloid culture. Results are expressed as number of colonies (CFU-GM and BFU-E) obtained per 1000 seeded cells, **c**) Cells from each of the first 10 days of myeloid culture were plated in methylcellulose colony-forming assays supplemented with DMSO (as control) or 50 µM SIT. Results were expressed as the ratio of colony formation in SIT to control. A linear fit was extrapolated for each type of progenitor. A paired Student's t-test established a significant difference for the impact of SIT on CFU-GM formation.

We used SIT at a concentration of 50 µM after having shown dose response on CFU-GM formation between 25 µM and 100 µM at day 6 of the culture (**Figure 6a**).

All along the 10 days of the culture, the cloning efficiency diminished for both CFU-GM and BFU-E but caspase-3 inhibition showed a strong effect only on CFU-GM (**Figure 6b**), confirming our previous results. This effect correlated with the duration of the culture: the effect of caspase-3 inhibition on myeloid colony formation increased with increasing differentiation of the cells (p = 0.002) (**Figure 6c**) and could be due to the induction of apoptosis previously observed in liquid myeloid culture.

## Discussion

Caspases are cysteine proteases that are implied in cell death [17], [18]. Apart from its role in mediating apoptosis caspase-3 activation has in recent years been shown to induce differentiation of certain cell types [19], [20], [21], [22].

A role for caspase-3 activation in erythroid differentiation has been established yet its exact function and position in this process remain a matter of controversy. In this study, we confirm a major non-apoptotic role of caspase-3 in the erythroid differentiation pathway. We show that the effect of caspase inhibition is a reduction of overall erythroid development, comprising both expansion and differentiation, and we suggest that the decreased enucleation yield is a secondary effect of delayed maturation and increased cell death predominantly of mature cell stages. This effect could be observed when the inhibitor was added upstream in the early stages of erythroid development or directly to mature cells. Despite or perhaps due to the high concentrations of caspase-3 inhibitors employed here, cell death in these late stage erythroblasts may occur through an autophagic pathway, as high numbers of large vacuoles could be seen in both nucleated and enucleated cells at late stages of culture. Blocking caspase activation via inhibitors has been found to induce alternative cell death pathways such as autophagy or necrosis through an increased production of reactive oxygen species (ROS) [23]. Autophagic mechanisms are of particular importance in terminal differentiation stages of erythroid cells for the removal of mitochondria in so-called mitophagy [24], [25] and might become deregulated when concentrations of ROS are further increased and could thus explain the severe effect of SIT on late erythroblast and reticulocyte stages.

While caspase-3 inhibition resulted in an overall reduction of both expansion and differentiation in the erythroid system, the effects observed in the myeloid system were very different and suggest an alternative mechanism of action. Whereas erythroid differentiation is reduced when caspase-3 activity is inhibited, myeloid differentiation seemed to be accelerated. Similar observations have been reported for the lymphoid lineage in a murine Casp3^−/−^ model, where B cells in the bone marrow seemed to undergo maturation in an accelerated manner [26]. Various studies have demonstrated the involvement of caspase-3 in terminal erythroid maturation and shown a block of erythroid differentiation through the inhibition of caspase activation by different strategies. It has been shown that the addition of ZVAD to erythroid culture results in a blockage at the basophilic stage of differentiation. Using RNA interference others demonstrated that reducing caspase-3 activity yields a 50% reduction in cells that undergo enucleation with no change in the fraction of apoptotic cells, and that a substantial fraction of treated cells are unable to complete the transition from pronormoblasts to basophilic normoblasts. The authors also found that it is the activation of the Fas receptor on CD34^+^ cells that initiates erythroid cell differentiation via caspase activation [9] and such a role of Fas in inducing erythroid differentiation is supported by findings in the mouse model [27], [28]. However, these *in vitro* experiments were based on erythropoiesis models with a maximum enucleation yield of 30% [5], [8] and thus have a limited capability to reproduce the *in vivo* system and may lead to an overestimation of the effect that caspase activation has on cell enucleation. The culture system employed in this study presents the clear advantage of providing a definitive read-out system where nearly 100% enucleation is achieved. An effect of caspase inhibition on erythroid expansion as observed here has not been reported in previous studies, however, ZVAD treatment resulted in an 80% reduction of cell numbers compared to control in a previous report [8], suggesting a significant reduction of cell proliferation and/or high occurrence of cell death. To determine the mechanism by which caspase-3 inhibition decreased cell proliferation, we investigated the cell cycle distribution in erythroid inhibitor treated cultures and observed an accumulation of cells in G_2_/M of the cell cycle. While cells did not become fully arrested, the delayed progression through G_2_/M would increase the total cycle time, explaining at least in part the reduction of cellular expansion.

These results highlight a novel function of caspase-3 in the cell cycle regulation of erythroid development. Results implying an involvement of caspases in cell cycle regulation have been previously obtained for other tissue types: Hashimoto *et al* reported that the inhibition of caspases resulted in cell cycle arrest in G_1_ and G_2_/M phases in three different cells lines (HepG2, HeLa, and Jurkat cells) and concluded that caspase-3 plays a critical role in cell proliferation [29]. Cell cycle analysis of inhibitor-treated myeloid cultures showed that cell expansion was reduced by a very different mechanism in the myeloid lineage. In contrast to the erythroid model, no altered cell cycle progression was observed but a high number of apoptotic cells were detected, thus indicating cell death as primary cause for the reduced cell yield. Cell cycle progression is tightly regulated by cyclins and cyclin-dependent kinases and may be delayed or blocked through an upregulation of cyclin-dependent kinase inhibitors such as p21. In late stage erythroid maturation GATA-1 coordinates cell differentiation with cell cycle arrest in G_1_ via activation of p21 [30], [31]. The delay of erythroid growth and differentiation and the increase in cell death under caspase-3 inhibition that we show here, suggest the involvement of major erythropoietic regulators such as transcription factor GATA−1.

The reduced cell expansion we observed in both myeloid and erythroid cultures initially suggested that caspase-3 activation acts in an early uncommitted or common precursor cell that possesses both myeloid and erythroid potential and led us to investigate at which stage cells became sensitive to caspase-3 inhibition. An activation of caspase-3 that is not associated with apoptosis has already been established in CD34^+^ HSC [5], [32], [33]. The erythroid colony forming potential of CD34^high^ cells was found to be unaffected in our model and showed hardly any changes up to day 4 in erythroid culture. Myeloid colony formation, however, was reduced in SIT-treated CD34^high^ HSC as early as day 0. These findings are in agreement with previous data, which also reported unaltered erythroid colony formation of Fas siRNA treated CD34^+^ cells [9] but did not investigate myeloid colony forming potential.

The strong effect of SIT on erythroid colony-formation after day 4 in erythroid culture therefore pointed to a more mature erythroid progenitor. By analysing the clonogenicity of a committed erythroid culture (7 days of culture with EPO, IL-3 and SCF) sorted into C36^+^/CD34^−^ and CD36^+^/CD34^+^, we were able to determine a susceptibility of late BFU-E and CFU-E to caspase inhibitor treatment, while early BFU-E contained in freshly isolated CD34^+^ cells had remained unaffected. A system lacking EPO in which immature erythroid progenitors (early BFU-E) are able to survive but are prevented from differentiating confirmed that early BFU-E clonogenicity remained unaltered, thus showing early BFU-E to be insensitive to caspase-3 inhibition.

Multiple roles for caspase-3 in erythroid development are highly likely as numerous proteins possess caspase recognition sites, and a role for caspase activation in early lineage development as we report here, does not rule out further functions during late erythroid cellular re-organization. Concentrations of caspase-3 molecules have been estimated at 2.1×10^4^ molecules per cell in unstimulated HeLa [34] cells or up to 1.6×10^6^ molecules per cell for certain cancer cell lines [35], and in most cell culture systems 2–10 µM of caspase inhibitor suffice to inhibit cell death after apoptotic stimulation. Concentrations of 50 µM of caspase-3 inhibitor used in our study amount to 3×10^10^ molecules per cell at a cell concentration of 1×10^6^ cells/ml, an amount largely in excess of what should be needed to inhibit caspase activation even if inhibitor uptake by the cell was incomplete. Such discrepancy could be explained by a transient but frequent short-term activation of caspases in a signalling role which does not result in activation of the complete apoptotic caspase cascade but involves a rapid turn-over of caspases by the ubiquitin-proteasome pathway [36].

This study illustrates the value of an erythropoiesis model that can fully recapitulate *in vivo* erythropoiesis including full enucleation in dissecting the regulatory processes that underlie erythropoiesis. We report that the role of caspase-3 activation as a signalling function in erythroid differentiation begins at the late BFU-E stage and suspect a main role in pathways responsible for the expression of erythroid genes and of anti-apoptotic genes not only in the erythroid but also the myeloid lineage such as the GATA-1 signalling pathway. As it was found that caspase activation occurred prior to the basophilic stage, caspase-3 thus seems to target cells that strongly express the EPO receptor (EPO-R) [37]. The expression of EPO-R gradually increases between mature BFU-E and CFU-E making these progenitors highly sensitive to EPO. These data thus fit within the pattern of action of caspases in which EPO dependent cells (i.e. cells expressing EPO-R) are rescued from apoptosis through the recruitment of the chaperone molecule HSP70 that prevents GATA-1 cleavage [38]. Subsequent proteomic and transcriptomic analyses should permit an identification of the actual key molecules involved in such a caspase-3 signalling pathway that appears to be essential for erythroid lineage development.
